# Long term outcome and quality of life after open incisional hernia repair - light versus heavy weight meshes

**DOI:** 10.1186/1471-2482-11-25

**Published:** 2011-09-14

**Authors:** Roland Ladurner, Costanza Chiapponi, Quirin Linhuber, Thomas Mussack

**Affiliations:** 1 Department of Surgery Innenstadt, Ludwig-Maximilian-University of Munich, Nussbaumstr. 20. 80336 Munich, Germany

**Keywords:** incisional hernia repair, heavy weight, low weight polypropylene mesh, quality of life

## Abstract

**Background:**

Mesh repair of incisional hernia is superior to the conventional technique. From all available materials for open surgery polypropylene (PP) is the most widely used. Development resulted in meshes with larger pore size, decreased mesh surface and lower weight. The aim of this retrospective non randomized study was to compare the quality of life in the long term follow up (> 72 month) after incisional hernia repair with "light weight"(LW) and "heavy weight"(HW) PP meshes.

**Methods:**

12 patients who underwent midline open incisional hernia repair with a HW-PP mesh (Prolene^® ^109 g/m^2 ^pore size 1.6 mm) between January 1996 and December 1997 were compared with 12 consecutive patients who underwent the same procedure with a LW-PP mesh (Vypro^® ^54 g/m^2^, pore size 4-5 mm) from January 1998. The standard technique was the sublay mesh-plasty with the retromuscular positioning of the mesh. The two groups were equal in BMI, age, gender and hernia size. Patients were routinely seen back in the clinic.

**Results:**

In the long term run (mean follow up 112 ± 22 months) patients of the HW mesh group revealed no significant difference in the SF-36 Health Survey domains compared to the LW group (mean follow up 75 ± 16 months).

**Conclusions:**

In this study the health related quality of life based on the SF 36 survey after open incisional hernia repair with light or heavy weight meshes is not related to the mesh type in the long term follow up.

## Background

Hernia repair is one of the most common surgical operations with more than 50.000 incisional hernia repaired every year in Germany. Patient seek surgical repair because of physical discomfort and aesthetic reasons. Both impair their quality of life. Unfortunately some patients complain of abdominal discomfort also after the operation. Despite the surgical technique one possible factor is the type of mesh used. Polypropylene (PP) is the most widely used mesh material for hernia repair. PP meshes show a high stretch and tensile strength, five times higher than the maximal physiologic stress. The extent of the scar tissue induced by the mesh depends on the amount and structure of the incorporated material and is responsible for the abdominal wall compliance [1,2]. In 20% of the cases heavy weight (HW) and small pore size PP meshes caused a reduction of the abdominal wall mobility ("stiff abdomen")[3,4]. This complication was associated with chronic abdominal pain. As a consequence macropore light weight (LW) PP meshes strong enough to resist maximal physiologic stress of the abdominal wall were developed [5]. This development resulted in a reduction of the chronic pain [1,6,7]. Whether macropore LW-PP meshes have also a beneficial effect on the life quality of patients in the long term outcome after open incisional hernia repair is still unclear. The objective of this study was therefore to assess the health related quality of life (HrQoL) and the long term outcome of patients with open incisional hernia repair using HW- versus LW-PP meshes.

## Methods

### Characteristics of the study group

33 patients with midline open incisional hernias were operated with a heavy weight mesh (Prolene^® ^108.5 g/m^2 ^pore size 1.6 mm, Ethicon, Nordersted; Germany) between January 1996 and December 1997 and were eligible for this study. Unfortunately only 12 of them could be followed up over the years. For the comparison the next 12 consecutive patients operated with a light weight mesh (Vypro^® ^54 g/m^2^, pore size 4-5 mm, Ethicon, Nordersted, Germany) after January 1998 were enrolled in the study. We excluded incarcerated, lateral or parastomal hernias, hernia repair not using mesh replacement and hernia repair performed with another procedure (laparoscopic hernia repair, onlay and inlay mesh-plasty).

The standard technique consisted of laparotomy, adhaesiolysis, hernia sac resection, closure of the posterior rectus fascia and retromuscular positioning of the PP mesh. Mesh size was chosen so that the margin extended beyond the margin of the defect throughout the defect's entire circumference. The mesh overlap was at least 5 cm. Mesh fixation was performed with interrupted PP 3/0 sutures in the midline and at the border of the mesh. The lateral mesh fixation occurred along the margin of the rectus muscle. By doing this the intercostal nerve branches were carefully preserved to avoid muscular atrophy. The anterior rectus sheath was closed with a continuous PDS 2/0 running suture loop.

Patients were routinely seen back in the clinic 3, 6 and 12 months postoperatively, then yearly thereafter. The standard follow up consisted of anamnesis, physical examination and ultrasound or MRI, when needed (anamnesis and physical examination suggestive of hernia recurrence or intestinal adhesions). The follow up could be completed in all 24 cases.

### Life Quality (SF36)

We used the SF-36, a multidimensional questionnaire composed of 36 items to determine the health related quality of life [8]. The SF36 health survey consists of eight different health quality domains: physical function (10 items), role limitations due to physical functions role (4 items), bodily pain (2 items), general health (5 items), vitality (4 items), social function (2 items), role limitations due to emotional function (3 items), mental health (5 items). The results from each scale vary from 0 to 100 (worst to best possible health status). Additionally, the SF-36 Physical Component Summary (PCS), and the Mental Component Summary (MCS) scales were determined, ranging from zero (lowest well-being) to 100 (highest well-being) [9,10]. The SF 36 Health survey scores were compared with the age-stratified German population. The questionnaire evaluates the negative health aspects (disease or illness) and the positives aspects (well being). The generic SF-36 was chosen instead of more specific scales like the CCS (Carolina Comfort Scale) [11] because it is the gold standard for measuring quality of life and it is validated in German language [12].

### Statistical analysis

All data are presented as means and standard deviation. Differences between the intervention groups (HW vs LW PP meshes) were tested for significance using the unpaired t test for quantitative parametric variables and the Mann-Withney rank sum test for quantitative non parametric variables. The comparisons were performed using STATVIEW 4.5 software (Abacus Concepts, Berkeley, CA). Significance levels were set as p < 0.05.

## Results

### Patient characteristics

**Figure 1:**
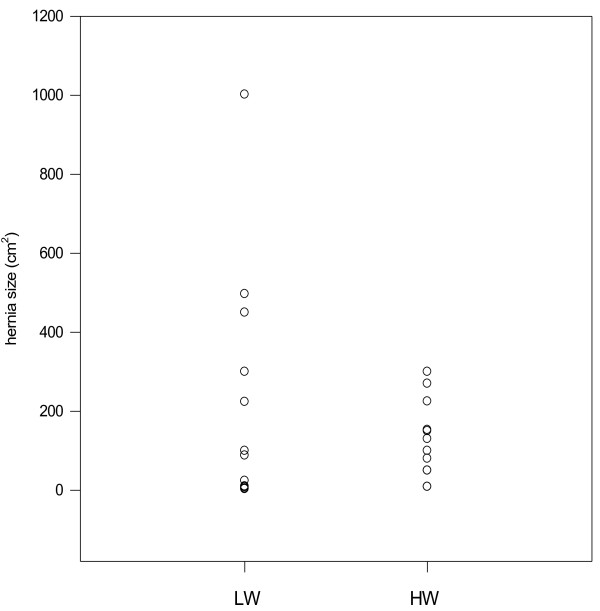
**Hernia size of the HW and LW-group**.

12 patients (mean age 57.3 ± 11.8, range 24-80 years) underwent sublay hernia repair with HW-PP meshes between January 1996 and December1997. The body mass index (BMI) was 29.8 ± 3.7 kg/m^2 ^and the hernia size was 127.2 ± 97.2 cm^2^. From January 1998 all patients received sublay hernia repair with a LW PP mesh. 12 consecutive patients (mean age 58.3 ± 11.1 years, range 28-82 years) were enrolled in the survey. The body mass index (BMI) was 28.7 ± 3.5 kg/m^2 ^and the hernia size 226 ± 301.5 cm^2^. The point plot graph shows the hernia size of the two groups (Figure 1).

There were no significant difference in age, gender, body mass index, hernia size, patient related risk factors, operation time and the length of the hospital stay (Table 1). As patient related risk factors were considered obesity (BMI > 30 kg/m^2^), diabetes mellitus, steroid therapy, nutritional deficiencies, renal impairment, chemotherapy, smoking, chest problems and hepatic cirrhosis [13]. Comorbidities were also comparable between the groups (Table 2).

**Table 1:** Demographic data

	LW mesh (Vypro^®^)	HW mesh (Prolene^®^)	p
number of patients	12	12	
sex ratio (male:female)	9:3	8:4	
age (year)	58.3 ± 11.1	57.3 ± 11.8	0.840
number of risk factors	1.8 ± 0.7	2.3 ± 1.2	0.318
BMI (kg/m^2^)	28.7 ± 3.5	29.8 ± 3.7	0.462
hernia size (cm^2^)	226.0 ± 301.5	127.2 ± 97.2	0.292
operation length (min)	105.0 ± 41.8	110.4 ± 23.5	0.699
hospital stay (d)	10.4 ± 3.8	6.8 ± 5.5	0.078

Data for the LW light weight and HW heavy weight group are presented as mean ± standard deviation. Differences were considered significant at p < 0.05.

**Table 2:** Comorbidities

	LW mesh (Vypro^®^)	HW mesh (Prolene^®^)
number of patients	12	12
cancer	3	4
asthma/COPD	3	3
heart disease	2	1
diabetes	1	2
back pain	1	-

### Long term follow up

The follow up could be completed in all 24 cases and was significant longer in the HW group (HW 112 ± 22 months; LW 75 ± 16 months).

In no case a hernia recurrence occurred. Two patients of the LW-group complained of low dragging pain during physical activity but no pain at rest (follow up 18 and 36 months respectively), whereas two patients of the HW-group reported on abdominal pain at rest as well as in motion (16.6%). One of these patients had a "stiff abdomen" (follow up 31 month), the other had no alteration of the abdominal wall mobility (follow up 89 month).

In these four patients (2 HW and 2 LW) ultrasound and MRI imaging studies were carried out. The polypropylene mesh was not visible in any of these cases. The abdominal wall in the patient with the "stiff abdomen" (HW group) showed a reduced mobility in the functional cine MRI study and an asymmetric fatty atrophy of the rectus muscle. In the other three patients (1 HW and 2 LW) no changes of the abdominal wall mobility or morphology could be detected. In all four patients intraabdominal adhesions could be identified, however these did not correlate with the mesh type.

Nevertheless in none of the eight domains of the SF 36 health survey a significant difference could be found. The SF36 Survey therefore showed no significant worse or better outcome for one mesh type in the long term follow up. The postoperative health-quality domains were lower than the scores of the age-stratefied healthy German population. (Table 3 and 4).

**Table 3:** SF 36 health survey

	LW mesh (Vypro^®^)	HW mesh (Prolene^®^)	p	German normal population
physical functioning	64.2 ± 26.5	71.3 ± 33.3	0.570	83.7 ± 19.5
physical role functioning	50.0 ± 46.5	45.8 ± 45.0	0.826	80.6 ± 31.9
bodily pain	62.3 ± 35.0	67.3 ± 31.9	0.718	72.7 ± 27.3
general health perceptions	50.6 ± 26.5	49.3 ± 21.5	0.894	61.0 ± 19.1
vitality	48.3 ± 13.4	51.3 ± 10.3	0.555	61.2 ± 17.3
social role functioning	74.0 ± 22.9	70.8 ± 21.5	0.734	86.8 ± 18.0
emotional role functioning	47.2 ± 48.1	77.8 ± 32.8	0.084	88.9 ± 26.9
mental health	60.3 ± 29.3	70.3 ± 12.4	0.288	72.4 ± 16.1

The first four domains describe the physical, the last four the mental health status. The results from each scale vary from 0 to 100 (worst to best possible health status). The scores are compared to the age-stratified German population (Bullinger M, Kirchberger I. S36 Fragebogen zum Gesundheitszustand Holzgreve 1998)

Data for the LW light weight and HW heavy weight group are presented as mean ± standard deviation. Differences were considered significant at p < 0.05.

**Table 4:** Physical and mental component summary

	LW mesh (Vypro^®^)	HW mesh (Prolene^®^)	German normal population
PCS	47.8 ± 7.5	49.4 ± 7.3	47.9 ± 9.7
MCS	46.6 ± 8.5	48.8.8 ± 10.0	51.16 ± 8.1

PCS (physical component summary) and MCS (mental component summary) in comparison to the age-stratified German population. (Bullinger M, Kirchberger I. S36 Fragebogen zum Gesundheitszustand Holzgreve 1998)

## Discussion

PP is one of the most widely used meshes in abdominal wall surgery. It elicits an intense desmoplastic reaction in tissue, accompanied initially by serous exudation and resulting eventually in the formation of a sheet of scar that uses the mesh as a scaffold for its formation [7,14]. This mesh integration process in the abdominal wall and extent of scar tissue are regulated by the amount and structure of the incorporated material. The heavier the mesh weight and the smaller the size of the mesh pores are, the greater the resulting amount of scar tissue. The wound healing process causes a contraction of the mesh about 40% [15]. A high inflammatory activity as in the wake of a high amount of foreign material can increase this rate up to 90% [1]. Moreover the tissue reaction is not uniform but an individual patient depending factor [16].

The initial deployment of abdominal wall meshes developed meshes with small pore size and accordingly heavy weight. The clinical consequence was an impairment of the abdominal wall compliance: so 20-38% of the patients complained of reduced flexibility of the abdominal wall ("stiff abdomen") [3,4,17]. Since chronic pain has direct impact on most of the daily activities, it's an important aspect in the outcome of hernia surgery [18]. So the health related quality of life (HrQoL) has been receiving increasingly significance in the outcome evaluation of surgical treatments in the last years [19-26].

To reduce the incidence of abdominal stiffness and chronic pain, macropore light weight PP meshes strong enough to resist maximal physiologic stress of the abdominal wall were developed [5]. The aim of this study was to investigate whether the mesh type (LW vs HW) has an impact on the "quality of life" in the long term follow up (> 72 months) after incisional hernia repair. Hypothesis was that the use of LW meshes lead to less chronic pain and therefore to a better physical, psychosocial and social well-being compared to patients with HW-meshes.

As an instrument to investigate this question the SF-36 Health Survey was used. This is a well validated generic health status measure as opposed to one that targets a specific age disease or treatment group [10,27,28]. The questionnaire is made of up eight-scales. Four of them describe the physical and four the mental health status. The SF 36 Health Survey has been translated in more than 50 languages and adjusted to the cultural variations [24,29]. For hernia patients no specific HRQoL questionnaires are available.

In our study two patients of the LW-group complained about low dragging pain during physical activity. The pain did not persist at rest. Two patients of the HW-group reported on abdominal pain at rest as well as in motion (16.6%). The reported intensity of the pain was lower in the LW-group. However the SF36 Survey showed no significant difference in the quality of life of patients operated with a LW -or a HW -mesh in the long term follow up.

This lack of difference in the quality of life of the two groups is an interesting finding. In fact most studies investigating the life quality of patients undergoing inguinal hernia repair showed significant better results for LW meshes [30].

This is probably due to the fact that requirements for inguinal and ventral open hernia repair are different. The quality and the causes of pain following surgery are also different in inguinal and incisional hernia repair [31-33]. As a complication of inguinal hernia repair patients suffer mostly of neuropathic pain [32,34]. This is due to nerve compression caused by nerve injury during surgery (suture, tacks, mesh) or by perineural fibrosis induced by the incorporated mesh. The key to avoid chronic neuropathic pain is the preservation of the nerves during surgery beside the choice of an appropriate mesh material [35].

After open incisional hernia repair dominates non neuropathic somatic pain, caused by mechanic pressure of the folded mesh or scar tissue. This pain condition is influenced by the mesh elastic properties and the amount and structure of the material used for repair [31-33]. The mesh type and structure are therefore the main reason for the abdominal wall compliance and pain in the postoperative period.

An explanation for the lack of difference in the quality of life of patients operated with a more elastic and lighter material compared to those operated with heavier and stiffer material might be that chronic pain did not have the expected influence on the daily activity of the patients.

Nikkolo et al arrived to a similar conclusion in a randomised trial comparing LW and HW meshes for inguinal hernia repair [36]. Also Conze et al found in a prospective randomized clinical trial comparing lightweight composite mesh with polyester or polypropylene mesh no difference in the SF 36 physical function scores or daily activity for the first 24 month after the operation. In this study the SF36 scores between 4 and 24 months showed no further improvement. These findings were also irrespective of the mesh type [37].

The mean age of patients undergoing incisional hernia repair was 57 ± 11 and 58 ± 11 years. The health perception changes with increasing age. So despite poorer role and physical function elderly patients have similar global health perception compared with younger individuals [38].

Another important aspect is that this study compares the quality of life following open incisional hernia repair after a longer period of time (112 ± 22 and 75 ± 16 months) in opposition to most studies with a follow up of 6 to 12 months. Probably patients get used to the increased abdominal wall stiffness caused in the long term. However two patients of the heavy weight group complained of chronic pain as opposed to the low weight group where two patients complained of discomfort.

More generally the HrQoL of patients undergoing incisional hernia repair (no matter if with low or heavy weight mesh) is worse than that of the healthy population in the same age. There are several possible explanations for this. The comorbidities (cancer, heart disease, diabetes, back pain) might have influence on the quality of life. Some of those diseases or their treatments (diabetes, COPD, corticosteroid-/chemotherapy) are know to be risk factors for hernia formation. On the other hand patients with chronic disease tend to downscale their expectations for life and feel satisfied as long as they can stabilize their condition and be free from complication and aggravation [39].

The study has several limitations. First the group size is small. Starting with January 1998 heavy weight meshes were no longer used in our department. Additionally due to the long term follow up also the drop out rate was high. Therefore it was not possible to include more patients in this study. This reduces the power of the statistic.

Second limit is the different length of observation of the groups. However the shortest median observation time is 75 months. In our experience patients symptoms do not change significantly more than 5 years postoperatively.

Another limitation is the method of measuring the quality of life. A hernia specific scale would be preferable. Such a validated instrument though was not available at the time of the study and does not exist up to now in German language. For this reason the SF-36 was used.

## Conclusions

In this study the health related quality of life based on the SF 36 survey after open incisional hernia repair with light or heavy weight meshes is not related to the mesh type in the long term follow up.

## Competing interests

The authors declare that they have no competing interests.

## Authors' contributions

RL and TM conceived this study, RL, QL and CC acquired and interpreted the data, RL and CC wrote the manuscript, TM revised the manuscript critically; all authors have given final approval of the version to be published.

## Pre-publication history

The pre-publication history for this paper can be accessed here:

http://www.biomedcentral.com/1471-2482/11/25/prepub

## References

[B1] Schumpelick V Klosterhalfen B Muller M Klinge U Minimized polypropylene mesh for preperitoneal net plasty (PNP) of incisional hernias Chirurg 1999 70 422 430 10.1007/s001040050666 10354839

[B2] Welty G Klinge U Klosterhalfen B Kasperk R Schumpelick V Functional impairment and complaints following incisional hernia repair with different polypropylene meshes Hernia 2001 5 142 147 10.1007/s100290100017 11759800

[B3] Read RC Barone GW Hauer-Jensen M Yoder G Properitoneal prosthetic placement through the groin. The anterior (Mahorner-Goss, Rives-Stoppa approach Surg Clin North Am 1993 73 545 555 8497802 10.1016/s0039-6109(16)46036-6

[B4] Klinge U Prescher A Klosterhalfen B Schumpelick V Development and pathophysiology of abdominal wall defects Chirurg 1997 68 293 303 10.1007/s001040050192 9206623

[B5] Klinge U Klosterhalfen B Conze J Limberg W Obolenski B Ottinger AP Schumpelick V Modified mesh for hernia repair that is adapted to the physiology of the abdominal wall Eur J Surg 1998 164 951 960 10029391 10.1080/110241598750005138

[B6] Cobb WS Kercher KW Heniford BT The argument for lightweight polypropylene mesh in hernia repair Surg Innov 2005 12 63 69 10.1177/155335060501200109 15846448

[B7] Bringman S Conze J Cuccurullo D Deprest J Junge K Klosterhalfen B Parra-Davila E Ramshaw B Schumpelick V Hernia repair: the search for ideal meshes Hernia 2010 14 81 87 10.1007/s10029-009-0587-x 20012333 PMC2815300

[B8] Bullinger M Assessment of health related quality of life with the SF-36 Health Survey Rehabilitation (Stuttg) 1996 35 XVII XXVII 8975342

[B9] Thaler K Dinnewitzer A Mascha E Arrigain S Weiss EG Nogueras JJ Wexner SD Long-term outcome and health-related quality of life after laparoscopic and open colectomy for benign disease Surg Endosc 2003 17 1404 1408 10.1007/s00464-002-8855-1 12802642

[B10] Ware JE Jr Sherbourne CD The MOS 36-item short-form health survey (SF-36). I. Conceptual framework and item selection Med Care 1992 30 473 483 10.1097/00005650-199206000-00002 1593914

[B11] Heniford BT Walters AL Lincourt AE Novitsky YW Hope WW Kercher KW Comparison of generic versus specific quality-of-life scales for mesh hernia repairs J Am Coll Surg 206 638 644 10.1016/j.jamcollsurg.2007.11.025 18387468

[B12] Bullinger M German translation and psychometric testing of the SF-36 Health Survey: preliminary results from the IQOLA Project. International Quality of Life Assessment Soc Sci Med 2008 41 1359 1366 10.1016/0277-9536(95)00115-n 8560303

[B13] El Khadrawy OH Moussa G Mansour O Hashish MS Prophylactic prosthetic reinforcement of midline abdominal incisions in high-risk patients Hernia 2009 13 267 274 10.1007/s10029-009-0484-3 19262985

[B14] Sergent F Desilles N Lacoume Y Tuech JJ Marie JP Bunel C Biomechanical analysis of polypropylene prosthetic implants for hernia repair: an experimental study Am J Surg 2010 200 406 412 10.1016/j.amjsurg.2009.09.024 20800718

[B15] Klinge U Klosterhalfen B Muller M Ottinger AP Schumpelick V Shrinking of polypropylene mesh in vivo: an experimental study in dogs Eur J Surg 1998 164 965 969 10029393 10.1080/110241598750005156

[B16] Schachtrupp A Klinge U Junge K Rosch R Bhardwaj RS Schumpelick V Individual inflammatory response of human blood monocytes to mesh biomaterials Br J Surg 2003 90 114 120 10.1002/bjs.4023 12520586

[B17] Schmidbauer S Ladurner R Hallfeldt KK Mussack T Heavy-weight versus low-weight polypropylene meshes for open sublay mesh repair of incisional hernia Eur J Med Res 2005 10 247 253 16033714

[B18] Poobalan AS Bruce J King PM Chambers WA Krukowski ZH Smith WC Chronic pain and quality of life following open inguinal hernia repair Br J Surg 2001 88 1122 1126 10.1046/j.0007-1323.2001.01828.x 11488800

[B19] Barrat C Seriser F Arnoud R Trouette P Champault G Inguinal hernia repair with beta glucan-coated mesh: prospective multicenter study (115 cases)--preliminary results Hernia 2004 8 33 38 10.1007/s10029-003-0156-7 13680304

[B20] Lawrence K McWhinnie D Jenkinson C Coulter A Quality of life in patients undergoing inguinal hernia repair Ann R Coll Surg Engl 1997 79 40 45 9038494 PMC2502599

[B21] Thaler K Dinnewitzer A Oberwalder M Weiss EG Nogueras JJ Wexner SD Assessment of long-term quality of life after laparoscopic and open surgery for Crohn's disease Colorectal Dis 2005 7 375 381 10.1111/j.1463-1318.2005.00769.x 15932562

[B22] Woodcock SA Watson DI Lally C Archer S Bessell JR Booth M Cade R Cullingford GL Devitt PG Fletcher DR Hurley J Jamieson GG Kiroff G Martin CJ Martin IJ Nathanson LK Windsor JA Quality of life following laparoscopic anterior 90 degrees versus Nissen fundoplication: results from a multicenter randomized trial World J Surg 2006 30 1856 1863 10.1007/s00268-005-0623-7 16983477

[B23] Zieren J Zieren HU Wenger F Muller JM Repair of inguinal hernia in the elderly. Results of the plug-and-patch repair with special reference to quality of life Chirurg 2000 71 564 567 10.1007/s001040051103 10875015

[B24] Korolija D Sauerland S Wood-Dauphinee S Abbou CC Eypasch E Caballero MG Lumsden MA Millat B Monson JR Nilsson G Pointner R Schwenk W Shamiyeh A Szold A Targarona E Ure B Neugebauer E Evaluation of quality of life after laparoscopic surgery: evidence-based guidelines of the European Association for Endoscopic Surgery Surg Endosc 2004 18 879 897 10.1007/s00464-003-9263-x 15108103

[B25] Poelman MM Schellekens JF Langenhorst BL Schreurs WH Health-related quality of life in patients treated for incisional hernia with an onlay technique Hernia 2010 14 237 242 10.1007/s10029-009-0619-6 20063109

[B26] Wassenaar E Schoenmaeckers E Raymakers J van der PJ Rakic S Mesh-fixation method and pain and quality of life after laparoscopic ventral or incisional hernia repair: a randomized trial of three fixation techniques Surg Endosc 2010 24 1296 1302 10.1007/s00464-009-0763-1 20033726 PMC2869434

[B27] McHorney CA Ware JE Jr Raczek AE The MOS 36-Item Short-Form Health Survey (SF-36): II. Psychometric and clinical tests of validity in measuring physical and mental health constructs Med Care 1993 31 247 263 10.1097/00005650-199303000-00006 8450681

[B28] McHorney CA Ware JE Jr Lu JF Sherbourne CD The MOS 36-item Short-Form Health Survey (SF-36): III. Tests of data quality, scaling assumptions, and reliability across diverse patient groups Med Care 1994 32 40 66 10.1097/00005650-199401000-00004 8277801

[B29] Alonso J Ferrer M Gandek B Ware JE Jr Aaronson NK Mosconi P Rasmussen NK Bullinger M Fukuhara S Kaasa S Leplege A Health-related quality of life associated with chronic conditions in eight countries: results from the International Quality of Life Assessment (IQOLA) Project Qual Life Res 2004 13 283 298 15085901 10.1023/b:qure.0000018472.46236.05

[B30] Khan LR Liong S de Beaux AC Kumar S Nixon SJ Lightweight mesh improves functional outcome in laparoscopic totally extra-peritoneal inguinal hernia repair Hernia 2010 14 39 45 10.1007/s10029-009-0558-2 19756914

[B31] Heise CP Starling JR Mesh inguinodynia: a new clinical syndrome after inguinal herniorrhaphy? J Am Coll Surg 1998 187 514 518 10.1016/S1072-7515(98)00215-4 9809568

[B32] Amid PK Causes, prevention, and surgical treatment of postherniorrhaphy neuropathic inguinodynia: triple neurectomy with proximal end implantation Hernia 2004 8 343 349 10.1007/s10029-004-0247-0 15290609

[B33] Cunningham J Temple WJ Mitchell P Nixon JA Preshaw RM Hagen NA Cooperative hernia study. Pain in the postrepair patient Ann Surg 1996 224 598 602 10.1097/00000658-199611000-00003 8916874 PMC1235436

[B34] Pappalardo G Frattaroli FM Mongardini M Salvi PF Lombardi A Conte AM Arezzo MF Neurectomy to prevent persistent pain after inguinal herniorraphy: a prospective study using objective criteria to assess pain World J Surg 2007 31 1081 1086 17420959 10.1007/s00268-006-7627-9

[B35] Alfieri S Rotondi F Di Giorgio A Fumagalli U Salzano A Di Miceli D Ridolfini MP Sgagari A Doglietto G Influence of preservation versus division of ilioinguinal, iliohypogastric, and genital nerves during open mesh herniorrhaphy: prospective multicentric study of chronic pain Ann Surg 2006 243 553 558 10.1097/01.sla.0000208435.40970.00 16552209 PMC1448978

[B36] Nikkolo C Lepner U Murruste M Vaasna T Seepter H Tikk T Randomised clinical trial comparing lightweight mesh with heavyweight mesh for inguinal hernioplasty Hernia 2010 14 253 258 10.1007/s10029-010-0630-y 20091327

[B37] Conze J Kingsnorth AN Flamnet JB Simmermacher R Arlt G Langer C Schippers E Hartely M Schumpelick V Randomized clinical trial comparing lightwight composite mesh with polyester or polypropylene mesh for incisional hernia repair Br J Surg 2005 92 1488 1493 10.1002/bjs.5208 16308855

[B38] Mangione CM Marcantonio ER Goldman L Cook EF Donaldson MC Sugarbaker DJ Poss R Lee TH Influence of age on measurement of health status in patients undergoing elective surgery J Am Geriatr Soc 1993 41 377 383 8463523 10.1111/j.1532-5415.1993.tb06944.x

[B39] Wang HM Beyer M Gensichen J Gerlach FM Health-related quality of life among general practice patients with differing chronic disease in Germany: Cross sectional survey BMC Public Health 2008 8 246 258 10.1186/1471-2458-8-246 18638419 PMC2515099

